# Tunnel versus medial approach in laparoscopic radical right hemicolectomy for right colon cancer: a retrospective cohort study

**DOI:** 10.1186/s12893-022-01491-5

**Published:** 2022-01-26

**Authors:** Xijie Zhang, Junli Zhang, Pengfei Ma, Yanghui Cao, Chenyu Liu, Sen Li, Zhi Li, Yuzhou Zhao

**Affiliations:** Department of General Surgery, Affiliated Cancer Hospital of Zhengzhou University, 127 Dong Ming Road, Zhengzhou, 450008 Henan China

**Keywords:** Colonic cancer, Tunnel approach, Laparoscopic surgery, Right hemicolectomy, Safety

## Abstract

**Purpose:**

This study aimed to explore the feasibility and safety of the tunnel approach in laparoscopic radical right hemicolectomy for colon cancer.

**Methods:**

From July 2016 to October 2018, a total of 106 consecutive patients with colon cancer who underwent laparoscopic radical right hemicolectomy at the Affiliated Cancer Hospital of Zhengzhou University were enrolled. The patients were stratified into either a tunnel approach (TA) (n = 56) group or traditional medial approach (MA) (n = 50) group according to the surgical technique performed. The baseline demographics, perioperative outcomes and oncologic outcomes were compared between the two groups.

**Results:**

The baseline characteristics did not differ between groups. The TA group had significantly less blood loss [20.0 (10.0–40.0) vs. 100 (100.0–150.0) ml, p < 0.001] and a shorter operation time [128.4 ± 16.7 vs. 145.6 ± 20.3 min, p < 0.001] than the MA group. The time to first flatus and postoperative hospital stay were similar [3.0 (2.0–4.0) vs. 3.0 (3–4.0) days, p = 0.329; 10.4 ± 2.6 vs. 10.7 ± 3.0 days, p = 0.506] between the two groups. The conversion to laparotomy and complication rates were similar between groups (0 vs. 6.0%, p = 0.203; 14.3% vs. 18.0%, p = 0.603, respectively). No treatment-related deaths occurred in either group. The TA group did not have significantly better survival outcomes than the MA group (p = 0.372).

**Conclusions:**

The TA seems to allow for more favourable results in terms of blood loss and operative time than the MA, with similar results regarding time to first flatus, hospital stay, postoperative complication rate, conversion rate and oncologic outcomes; moreover, the TA is easier for beginners to master.

## Introduction

Laparoscopic right hemicolectomy was first recommended by Jacobs et al. [1] in the 1990s, and laparoscopic radical right hemicolectomy has become the standard procedure for the treatment of right-sided colon cancer, achieving better short-term outcomes and comparable effectivity and safety to laparotomy [2–4]. Hohenberger et al. [5] also recommended the concept of complete mesocolic excision (CME) with high arterial ligation in 2009. Recent studies have confirmed that CME can lead to more thorough lymph node dissection and better oncological outcomes without increasing the risk of complications [6–8].

Multiple studies have shown that the various approaches achieve different advantages for laparoscopic right hemicolectomy. At present, the medial approach (MA) is the most widely accepted approach according to the “no-touch” principle and its safety profile [9–13]. However, the MA in laparoscopic right hemicolectomy is a demanding procedure with a steep learning curve and has a high rate of conversion to laparotomy, mainly because of anatomic complexities and high level of variation in the right colonic vessels [14, 15]. In reference to the different aforementioned surgical approaches, we improved the caudal approach then explored the tunnel approach (TA) to perform CME based on the idea of an “easier surgery”. TA begins by dissociating the attachment of the ileocecal region and the retroperitoneum and forms a tunnel upward through Toldt’s gap, making it easier to expose the superior mesentery vessels. Previous studies have shown that TA has achieved satisfactory clinical results [16]. This study aimed to explore the surgical feasibility and safety of the TA in comparison to the MA in laparoscopic right hemicolectomy.

## Methods

### Search strategy

A total of 106 consecutive patients with right colonic cancer who underwent laparoscopic radical right hemicolectomy were enrolled in the Affiliated Cancer Hospital of Zhengzhou University from July 2016 to October 2018. At the early stage of developing the TA, it is not sure whether it would have clinical advantages. We intend to further promote this approach in the clinic, hence two different procedures were applied in our hospital in the same period. We explained to the patient and their families two kinds of operation procedures in the preoperative conversation. The patients were divided into the TA subgroup (n = 56) and traditional MA subgroup (n = 50) according to the procedure determined by their personal wishes. The inclusion criteria were as follows: (1) diagnosis of right-sided colonic cancer with a clinical stage of I–III based on preoperative colonoscopy and abdominal enhanced computed tomography (CT); (2) single tumour in the ileocecum, ascending colon, hepatic flexure or right transverse colon; (3) tumour size found to be ≤ 10 cm during intraoperative laparoscopic exploration; and (4) no invasion into the adjacent organs. Patients with a diagnosis of colonic adenocarcinoma were considered. The exclusion criteria were as follows: (1) previous neoadjuvant therapy; (2) patients with distant metastases confirmed by imaging; (3) preoperative symptoms of intestinal obstruction; and (4) could not tolerate laparoscopy due to other organ dysfunction. TNM staging was performed using the National Comprehensive Cancer Network (NCCN) guidelines for colon cancer (v1. 2019). [17].

### Operative approach

#### TA group: 56 patients underwent the tunnel approach

##### First: separating the attachment of the terminal ileum from the posterior peritoneum

**Fig. 1:**
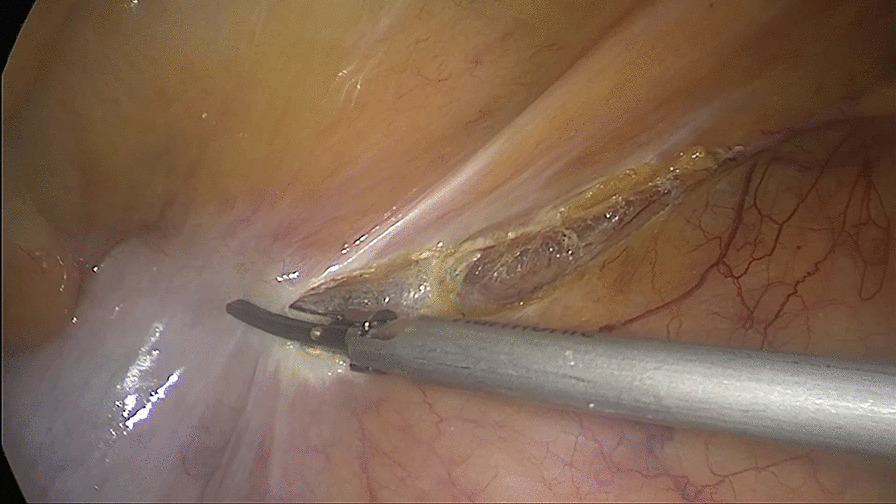
Separating the attachment of the terminal ileum from the posterior peritoneum

The patient was placed in the Trendelenburg position at 15–30°, and the body was tilted 15° to the left to allow the small intestine to fall to the left side of the abdominal cavity. The terminal ileum was separated from the posterior peritoneum to reach the lateral peritoneum of the ileocecum and the superior mesenteric artery trunk on the medial side (Fig. 1).

##### Second: dissociate the tissue cephalad along the Toldt’s gap

**Fig. 2:**
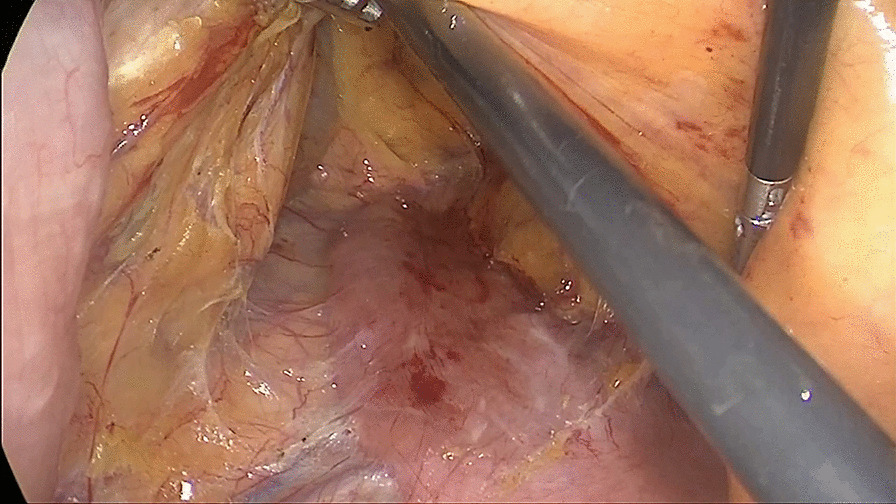
Dissociating the anterior tissue of Toldt’s gap

Toldt’s gap was entered through an incision. The Toldt’s gap was dissociated cephalad, and then the right mesocolon was separated from the retroperitoneum. The mesocolon of the right hemicolon was separately bluntly and sharply from the surface of the duodenum and pancreas head until the duodenal bulb was reached (Fig. 2).

##### Third: dissociating the hepatic flexure to the right until white line of Toldt

**Fig. 3:**
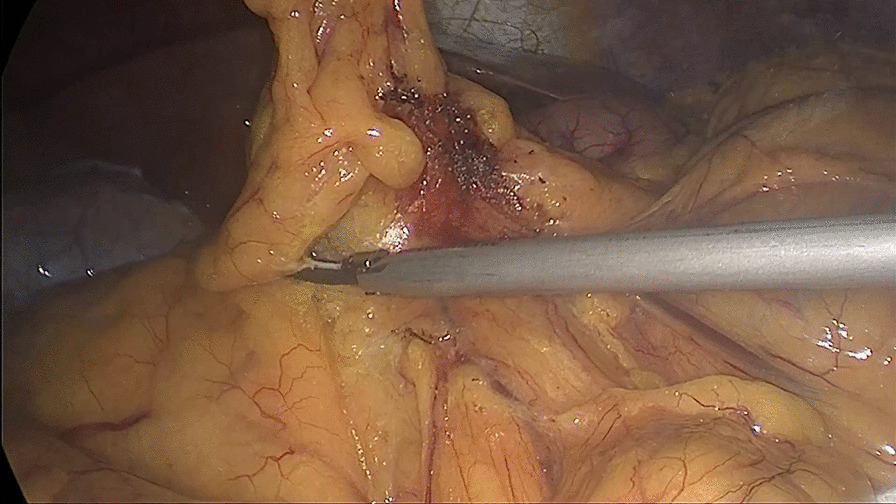
Dissociating the hepatic flexure to the right

The patient was placed in the dorsal elevated position at 15–30° and tilted 15° to the left to expose the omentum majus. The omentum majus was incised where the transverse colon was pre-resected. The hepatic flexure was dissociated to the right until reaching the white line of Toldt (the tissue was dissociated in or out of the gastro-omental vascular arch according to the location of the tumour: tissue was dissociated out of the arch without dissecting the No. 6 lymph nodes when the tumour was located at the ileocecum or ascending colon and in the arch close to the gastric wall; the No. 6 lymph nodes were dissected when the tumour was located at the hepatic flexure or transverse colon near the hepatic flexure) (Fig. 3).

##### Fourth: exposing and incising the blood vessels along the trunk of the superior mesenteric artery and vein

**Fig. 4:**
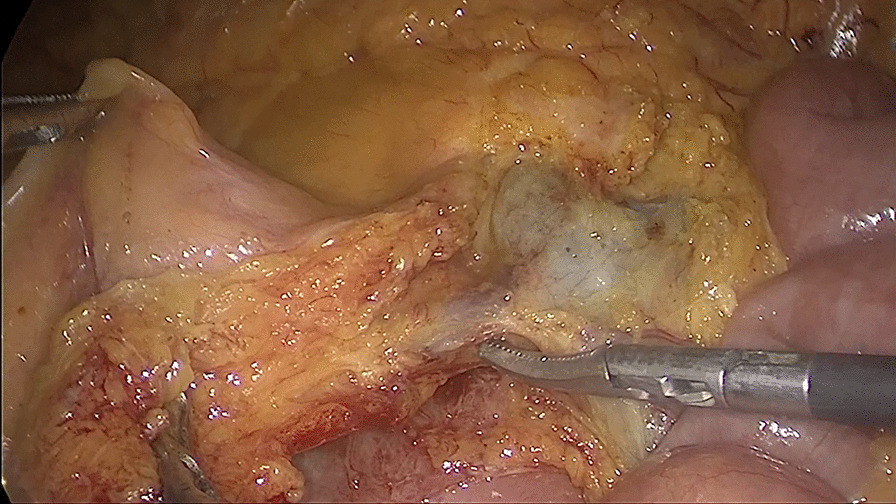
Exposing the trunk of the superior mesenteric vein

**Fig. 5:**
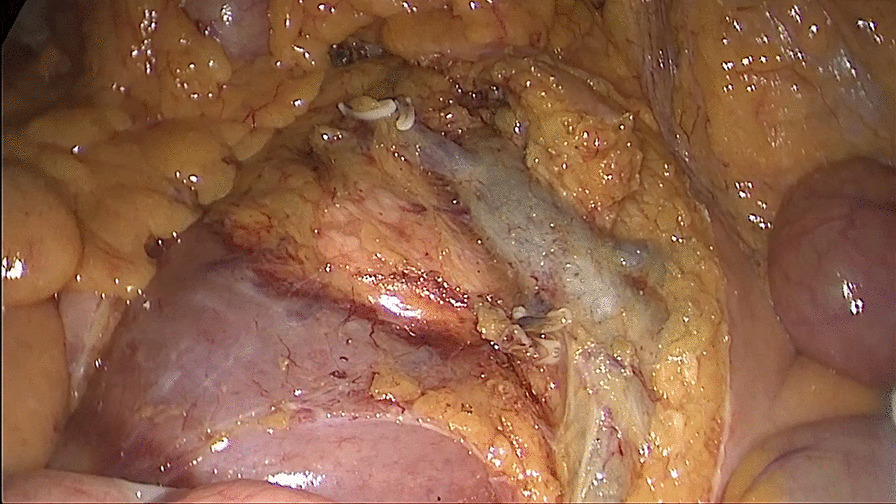
Completely exposing and dividing the vascular branches along the trunk of the superior mesenteric vessel

The ileocolic mesenteric avascular zone were incised below the ileocolic vessel, and the root of the ileocolic vessel was exposed from the right side of the superior mesenteric vein. Then, the ileocolic artery and vein, right colonic artery and vein, middle colonic artery (or its right branch) and vein and gastric colon vein trunk (or its colonic branch) were exposed and divided along the trunk of the superior mesenteric artery and vein (different patients may have the different vascular variations) (Figs. 4 and 5).

##### Last: removing the specimen completely and reconstructing the digestive tract

**Fig. 6:**
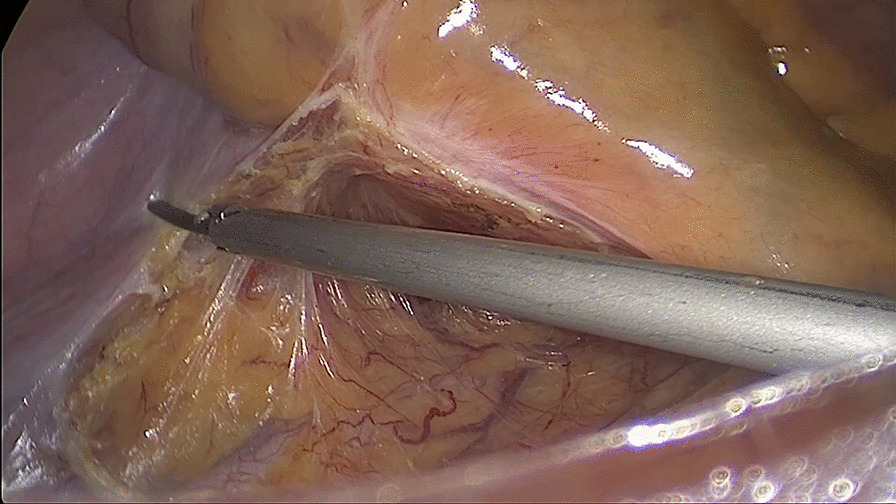
Dissociating the lateral peritoneum sharply along the paracolic sulcus of the ascending colon

The lateral peritoneum was dissociated sharply along the paracolic sulcus of the ascending colon, and then the specimen was removed completely. An approximately 6 cm incision was made 2 cm above the umbilicus. (The length of the incision depended on the size of the tumour). The incision was opened and protected with an incision protector, and an extracorporeal functional ileotransverse anastomosis was performed through the incision. Then, the abdominal cavity was irrigated with distilled water at 43 °C, and two abdominal drainage tubes were inserted prior to abdominal closure (Fig. 6).

### MA group: 50 patients underwent the traditional medial-to-lateral approach

#### Exposing and incising the mesenteric vessels

The ascending mesocolon was incised along the left side of the superior mesenteric vein at the root of the ileocolic vasculature to enter Toldt’s gap. Then, the anterior tissue was dissected along the superior mesenteric vein until reaching the lower edge of the pancreatic neck, crossing the horizontal segment of the duodenum and pancreatic uncinate. Then, the ileocolic artery and vein, right colonic artery and vein, middle colonic artery (or its right branch) and vein and gastric colon vein trunk (or its colonic branch) were exposed and divided along the trunk of the superior mesenteric artery and vein. To date, dissection of the right mesenteric vessel and surrounding lymph nodes has been completed.

#### Dissociating the mesocolon completely

The ascending mesentery was incised close to the right side of the superior mesenteric vein to enter Toldt’s gap. This, Toldt’s gap was dissected to the right until reaching the right white line of Toldt, dissected anterior until reaching the root of the transverse mesocolon, crossing the junction of the descending and horizontal duodenal segments, and dissected anteriorly until reaching the root of the ileal mesentery. Thus, the right colonic mesentery was completely separated from the retroperitoneum.

#### Dissociating the lateral peritoneum of the colon

The omentum majus was incised where the transverse colon was pre-resected, and the hepatic flexure was dissociated toward the right (with the same technique as that in the TA group). The right white line of Toldt was incised anteriorly from the hepatic flexure to the caecum, and the right colon and its corresponding mesentery were dissociated completely.

#### Removing the specimen completely and reconstructing the digestive tract

The operation was performed as described in the TA group.

### Statistical analysis

All statistical data were analysed with SPSS version 22.0 (IBM Corp., Armonk, New York, USA). The continuous variables are expressed as the mean ± standard deviation (SD) and compared using the t test or were expressed as the median [inter-quartile range (IQR)] and compared using the rank-sum test, according to whether the data fit a normal distribution. The chi-square test or Fisher’s exact test was used to compare categorical variables. We defined a p value < 0.05 as statistically significant.

## Results

### Clinicopathologic characteristics

The mean age of the patients was 57 years (29–84 years). A total of 55 patients were male, and 51 were female. In these 106 patients, the tumours were located in the ileocecum (n = 20, 18.9%), ascending colon (n = 56, 52.8%), hepatic flexure (n = 18, 17.0%) and right transverse colon (n = 12, 11.3%). According to NCCN guidelines for colon cancer (v1.2019), TNM staging was performed based on enhanced CT, revealing TNM stages of I (n = 13, 12.3%), II (n = 55, 51.9%) and III (n = 38, 35.8%). The histological types included highly differentiated adenocarcinoma (n = 2, 1.9%), moderately differentiated adenocarcinoma (n = 61, 57.5%), poorly differentiated adenocarcinoma (n = 35, 33.3%) and mucinous adenocarcinoma (n = 8, 7.5%). The baseline demographics were similar between the two groups (Table 1).

**Table 1:** Clinical characteristics of patients

Variable	TA group (n = 56)	MA group (n = 50)	χ^2^ or t test	*p* value
Gender, n (%)			χ^2^ = 0.641	0.423
Male	27 (48.2)	28 (56.0)		
Female	29 (51.8)	22 (44.0)		
Age (years), means ± SD	58.4 ± 12.0	56.0 ± 11.5	t = 1.019	0.310
BMI (kg/m^2^), means ± SD	23.0 ± 2.8	23.4 ± 2.7	t = 0.816	0.417
TNM stage, n (%)			Z = 0.144	0.886
I	6 (4.8)	7 (8.9)		
II	31 (66.7)	24 (51.1)		
III	19 (28.5)	19 (40)		
Location, n (%)			χ^2^ = 3.833	0.280
Ileocecus	14 (28.6)	6 (13.3)		
Ascending colon	29 (42.9)	27 (53.3)		
Hepatic flexure	7 (16.7)	11 (20)		
Right transverse colon	6 (11.8)	6(13.3)		
Differentiation, n (%)			χ^2^ = 0.065	0.996
Highly differentiated	1 (2.4)	1(2.2)		
Moderately differentiated	32 (52.4)	29(55.6)		
Poorly differentiated	19 (38.1)	16(33.3)		
Mucinous	4 (7.1)	4(8.9)		

### Operative outcomes

All 106 patients successfully underwent laparoscopic radical right hemicolectomy, and R0 resection was confirmed by postoperative pathology results. The mean operative time was significantly longer in the TA group than in the MA group (128.4 ± 16.7 vs. 145.6 ± 20.3 min, p < 0.001). Moreover, the volume of intraoperative blood loss was significantly lower in the TA group than in the MA group [20.0 (10.0–40.0) vs. 100 (100.0–150.0) ml, p < 0.001]. There was no significant difference in tumour size or lymph node yield between groups. Three patients (6.0%) required conversion to laparotomy in the MA group; the reasons were severe adhesions in one patient and uncontrolled intraoperative bleeding in two patients (Table 2).

**Table 2:** Perioperative results

Variable	TA Group (n = 56)	MA Group (n = 50)	χ^2^, t or Z	*p* value
Blood loss (ml), median (IQR)	20.0 (5.0–40.0)	100 (50.0–150.0)	Z = 7.137	< 0.001
Operation time (min), means ± SD	128.4 ± 16.7	145.6 ± 20.3	t = 4.784	< 0.001
Cancer size (cm), median (IQR)	4.0 (3.5–5.5)	5.0 (3.5–6.0)	Z = 1.490	0.136
Intraoperative conversion, n (%)	0	3 (6.0%)	χ^2^ = 1.620	0.203
Lymph node harvest, median (IQR)	29.5 (18.0–41.8)	26.0 (18–35)	Z = 0.874	0.382
The first flatus (days), median (IQR)	3.0 (2.0–4.0)	3.0 (3.0–4.0)	Z = 0.976	0.329
Postoperative hospitalization (days), means ± SD	10.4 ± 2.6	10.7 ± 3.0	t = 0.667	0.506
Complication, n (%)	8 (14.3%)	9 (18.0%)	χ^2^ = 0.271	0.603
Pneumonia	6	4		
Ileus	2	3		
Wound infection	0	1		
Anastomotic stenosis	0	1		

### Postoperative recovery

The time to first flatus (3.0 (2.0–4.0) vs. 3.0 (3.0–4.0) days, p = 0.329) and length of hospital stay (10.4 ± 2.6 vs. 10.7 ± 3.0 days, p = 0.506) were not significantly different between the TA group and MA group. The postoperative complication rate was similar between the two groups (eight patients (14.3%) vs. nine patients (18.0%), p = 0.603). One patient in the TA group (1.8%) experienced major complications (ileus), and two patients in the MA group (4.0%) experienced major complications (anastomotic stenosis in one patient and ileus in one patient) (p = 0.921). No mortalities occurred within 30 days after surgery in either group. Ninety-two patients were treated with adjuvant chemotherapy (Table 2).

### Survival outcomes

**Fig. 7:**
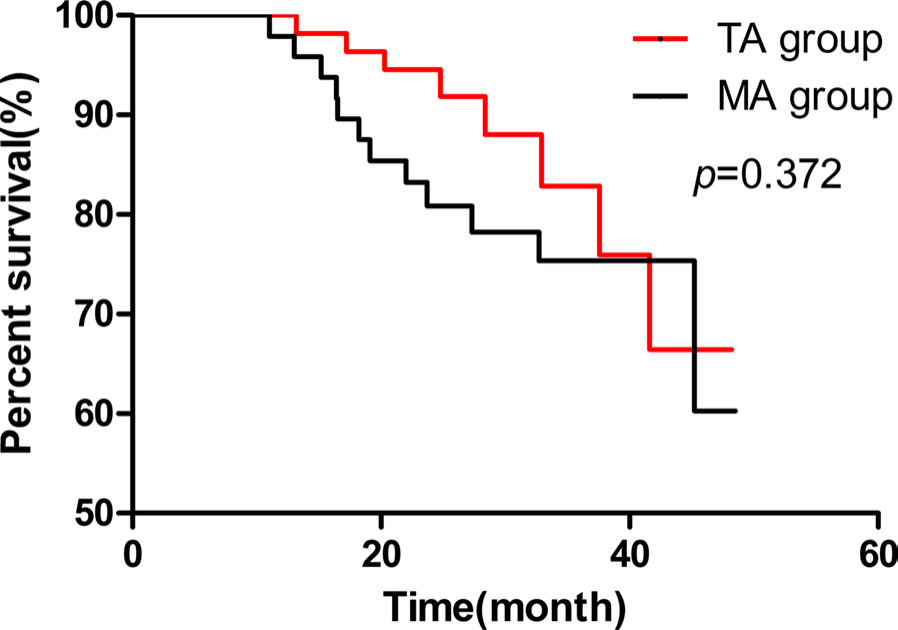
Overall survival rate of the TA and MA groups

One patient (1.8%) in the TA group and two patients (4.0%) in the MA group were lost to follow-up. Kaplan–Meier analysis showed that the TA group did not have a significantly longer survival duration (mean, 42.96; 95% CI 39.76–46.15 months) than the MA group (mean, 41.06; 95% CI 37.38–44.74) for patients of MA group (p = 0.372) (Fig. 7).

## Discussion

In the 1980s, JGR recommend that proximal and distal margins of the colonic tumor should be incised at least 5–10 cm long with corresponding mesenterium [18]. Hohenberger et al. [5] also recommended the concept of CME in 2009; since then, CME has been expected to become a standard procedure, as it is a novel concept for colectomy.

Laparoscopic right hemicolectomy can lead to less bleeding, less trauma, faster recovery of gastrointestinal function and shorter postoperative hospital stay than traditional laparotomy [19]. However, the laparoscopic approach has a steep learning curve [20]. Therefore, numerous studies have explored different surgical approaches in an attempt to find a better approach. Ding et al. showed that the medial approach is the preferable approach at present [21].

Kuzu et al. [22] showed that different variations of the right colonic vessels are an important reason for the long learning curve of the laparoscopic approach. For experienced surgeons, it is not difficult to completely expose the superior mesenteric vein, but this task is hard for beginners to master. In the medial approach, it is very difficult to dissect the root of the right gastroepiploic artery, which can cause haemorrhage and more serious consequences. The key factor during laparoscopic right hemicolectomy is successfully entering the anatomical plane quickly and accurately to expose the gastrocolic trunk. We created the tunnel approach based on various clinical procedures [23, 24]. This technique starts by dissecting the attachment between the ileocecal region and the retroperitoneum so that Toldt’s gap can be easily entered, regardless of their bodily form. This approach can also help beginners avoid dissociating superior mesenteric vessels at first. The anatomy is essentially converted from two-dimensional to three-dimensional, so the superior mesentery vessels are exposed more easily after the right mesentery is completely dissociated, which reduces the risk of bleeding and conversion rate and makes the approach easier for beginners to master.

In the present study, the TA group had significantly less intraoperative blood loss and a shorter operation time than the MA group. Moreover, the mean conversion rate and major postoperative complication rate were lower in the TA group than in the MA group. The reduced risks of severe complications and conversion are potential advantages of the TA in laparoscopic colectomy compared with the MA. However, the differences may be caused by the small sample size, and these findings need to be further confirmed in large-sample studies. The conversion rates in randomized controlled trials comparing laparoscopic colectomy with other approaches ranged from 0 to 18.1% [25, 26]. The reasons for conversion from laparoscopic colectomy for tumours included tumour invasion, abdominal adhesions, intraoperative bleeding, anatomical complexity and so on [27–29]. Tarnowski et al. [30] showed that the main reason for conversion was local tumour progression. In the present study, two of the three conversions were due to uncontrolled bleeding during dissection of the superior mesenteric artery, which is considered a complicated procedure in laparoscopic surgeries. The difference between the results of this study and previous studies may also be due to the insufficient sample size. There was no significant difference in overall survival between the two groups, which may also be caused by an insufficient follow-up time, so a longer follow-up is needed. The bottom-up suprapubic approach proposed by Petz et al. [31] for robotic CME in right colectomy is similar to our TA and also showed good clinical and oncologic results. Nevertheless, this approach ligates and incises the related branches of the superior mesenteric vessels directly after dissociating Toldt’s gap and then completely dissociates the transverse mesentery. Our method lifts the right mesentery completely before dissociating the branches of the superior mesenteric vein. Therefore, we believe that the tunnel method is more advantageous than the suprapubic approach, especially for obese patients.

The following points should be considered during the operation: (1) do not dissociate too deeply to avoid injuring the ureter and gonad vessels during the procedure; (2) after entering Toldt’s gap, an ultrasonic scalpel should be use to completely dissociate the anatomical plane close to the mesocolon; (3) primarily perform blunt dissociation and sometimes sharp dissociation with care to protect the duodenum; (4) when dissociating the liver flexure, avoid entering the Gerota fascia and prevent injury to the right kidney by putting gauze at the root of the transverse mesocolon.

Nonetheless, this study is subject to several limitations. First, observational and nonexperimental methods are inherent weaknesses of a retrospective design. Second, the findings may lack generalizability due to the relatively small number of cases. The long-term outcomes of randomized clinical trials or multicentre studies with a large number of cases are required for further confirmation of these results. Last, this study did not sufficiently assess the long-term outcomes due to an insufficient follow-up time.

In conclusion, this study suggests that the TA in laparoscopically assisted radical right hemicolectomy is a technically feasible and safe procedure. This approach has the advantages of a shorter operation time, less intraoperative blood loss, lower conversion and complication rates and shorter learning curve than the traditional MA in laparoscopic right hemicolectomy. Therefore, this new surgical approach is recommended for right hemicolectomy.

## Data Availability

The datasets analysed during the current study available from the corresponding author on reasonable request.

## Abbreviations

TA: Tunnel approach
MA: Medial approach
CME: Complete mesocolic excision
CT: Computed tomography
NCCN: National Comprehensive Cancer Network
SD: Standard deviation
IQR: Inter-quartile range

## References

[1] Jacobs M, Verdeja JC, Goldstein HS (1991). Minimally invasive colon resection (laparoscopic colectomy). Surg Laparosc Endosc.

[2] Clinical Outcomes of Surgical Therapy Study Group, Nelson H, Sargent DJ, Wieand HS, Fleshman J, Anvari M, et al. A comparison of laparoscopically assisted and open colectomy for colon cancer. N Engl J Med. 2004;350:2050–2059. 10.1056/NEJMoa032651. 10.1056/NEJMoa032651 15141043

[3] Colon Cancer Laparoscopic or Open Resection Study Group, Buunen M, Veldkamp R, Hop WC, Kuhry E, Jeekel J, et al. Survival after laparoscopic surgery versus open surgery for colon cancer: long-term outcome of a randomised clinical trial. Lancet Oncol. 2009;10:44–52. 10.1016/S1470-2045(08)70310-3. 10.1016/S1470-2045(08)70310-3 19071061

[4] Shin JK, Kim HC, Lee WY, Yun SH, Cho YB, Huh JW (2018). Laparoscopic modified mesocolic excision with central vascular ligation in right-sided colon cancer shows better short- and long-term outcomes compared with the open approach in propensity score analysis. Surg Endosc.

[5] Hohenberger W, Weber K, Matzel K, Papadopoulos T, Merkel S (2009). Standardized surgery for colonic cancer: complete mesocolic excision and central ligation—technical notes and outcome. Colorectal Dis.

[6] West NP, Hohenberger W, Weber K, Perrakis A, Finan PJ, Quirke P (2010). Complete mesocolic excision with central vascular ligation produces an oncologically superior specimen compared with standard surgery for carcinoma of the colon. J Clin Oncol.

[7] Bertelsen CA, Neuenschwander AU, Jansen JE, Wilhelmsen M, Kirkegaard-Klitbo A, Tenma JR (2015). Disease-free survival after complete mesocolic excision compared with conventional colon cancer surgery: a retrospective, population-based study. Lancet Oncol.

[8] Pedrazzani C, Lazzarini E, Turri G, Fernandes E, Conti C, Tombolan V (2019). Laparoscopic complete mesocolic excision for right-sided colon cancer: analysis of feasibility and safety from a single western center. J Gastrointest Surg.

[9] Li F, Zhou X, Wang B, Guo L, Wang J, Wang W (2017). Comparison between different approaches applied in laparoscopic right hemi-colectomy: a systematic review and network meta-analysis. Int J Surg.

[10] Matsuda T, Iwasaki T, Mitsutsuji M, Hirata K, Maekawa Y, Tanaka T (2015). Cranial-to-caudal approach for radical lymph node dissection along the surgical trunk in laparoscopic right hemicolectomy. Surg Endosc.

[11] Zou L, Xiong W, Mo D, He Y, Li H, Tan P (2016). Laparoscopic radical extended right hemicolectomy using a caudal-to-cranial approach. Ann Surg Oncol.

[12] Bergamaschi R, Schochet E, Haughn C, Burke M, Reed JF, Arnaud JP (2008). Standardized laparoscopic intracorporeal right colectomy for cancer: short-term outcome in 111 unselected patients. Dis Colon Rectum.

[13] Du S, Zhang B, Liu Y, Han P, Song C, Hu F (2018). A novel and safe approach: middle cranial approach for laparoscopic right hemicolon cancer surgery with complete mesocolic excision. Surg Endosc.

[14] Ye K, Lin J, Sun Y, Wu Y, Xu J, He S (2018). Variation and treatment of vessels in laparoscopic right hemicolectomy. Surg Endosc.

[15] Lee SJ, Park SC, Kim MJ, Sohn DK, Oh JH (2016). Vascular anatomy in laparoscopic colectomy for right colon cancer. Dis Colon Rectum.

[16] Zhang XJ, Zhang JL, Li S, Ma PF, Zhao YZ (2016). A tunnel approach in laparoscopically assisted radical right hemicolectomy—a video vignette. Colorectal Dis.

[17] National Comprehensive Cancer Network. Colon cancer (version 1.2019). https://www.nccn.org/professionals/physician_gls/pdf/colon.pdf. Accessed 15 Mar 2019.

[18] Japanese Research Society for Cancer of the Colon and Rectum, Dennosuke J. General rules for clinical and pathological studies on cancer of the colon, rectum and anus. Part I. Clinical classification. Jpn J Surg. 1983;13:557–73. 10.1007/bf02469505. 10.1007/BF02469505 6672390

[19] Chaouch MA, Dougaz MW, Bouasker I, Jerraya H, Ghariani W, Khalfallah M (2019). Laparoscopic versus open complete mesocolon excision in right colon cancer: a systematic review and meta-analysis. World J Surg.

[20] Kim J, Edwards E, Bowne W, Castro A, Moon V, Gadangi P (2007). Medial-to-lateral laparoscopic colon resection: a view beyond the learning curve. Surg Endosc.

[21] Ding J, Liao GQ, Xia Y, Zhang ZM, Pan Y, Liu S (2013). Medial versus lateral approach in laparoscopic colorectal resection: a systematic review and meta-analysis. World J Surg.

[22] Kuzu MA, İsmail E, Çelik S, Şahin MF, Güner MA, Hohenberger W (2017). Variations in the vascular anatomy of the right colon and implications for right-sided colon surgery. Dis Colon Rectum.

[23] Yamaguchi S (2010). Laparoscopic right hemicolectomy with intracorporeal anastomosis. Tech Coloproctol.

[24] Yang X, Wu Q, Jin C, He W, Wang M, Yang T (2017). A novel hand-assisted laparoscopic versus conventional laparoscopic right hemicolectomy for right colon cancer: study protocol for a randomized controlled trial. Trials.

[25] Waters PS, Cheung FP, Peacock O, Heriot AG, Warrier SK, O'Riordain DS (2020). Successful patient-oriented surgical outcomes in robotic vs laparoscopic right hemicolectomy for cancer—a systematic review. Colorectal Dis.

[26] Tekkis PP, Senagore AJ, Delaney CP, Fazio VW (2005). Evaluation of the learning curve in laparoscopic colorectal surgery: comparison of right-sided and left-sided resections. Ann Surg.

[27] Zarzavadjian le Bian A, Genser L, Denet C, Ferretti C, Laforest A, Ferraz JM, et al. Safety and feasibility of repeat laparoscopic colorectal resection: a matched case–control study. Surg Endosc. 2016;34:2120–6. 10.1007/s00464-019-06995-5. 10.1007/s00464-019-06995-5 31324972

[28] Ardu M, Bergamini C, Martellucci J, Prosperi P, Valeri A (2016). Colonic splenic flexure carcinoma: is laparoscopic segmental resection a safe enough oncological approach. Surg Endosc.

[29] Scotton G, Contardo T, Zerbinati A, Tosato SM, Orsini C, Morpurgo E (2018). From laparoscopic right colectomy with extracorporeal anastomosis to robot-assisted intracorporeal anastomosis to totally robotic right colectomy for cancer: the evolution of robotic multiquadrant abdominal surgery. J Laparoendosc Adv Surg Tech A.

[30] Tarnowski W, Uryszek M, Grous A, Dib N (2012). Intraoperative difficulties and the reasons for conversion in patients treated with laparoscopic colorectal tumors. Pol Przegl Chir.

[31] Petz W, Ribero D, Bertani E, Borin S, Formisano G, Esposito S (2017). Suprapubic approach for robotic complete mesocolic excision in right colectomy: oncologic safety and short-term outcomes of an original technique. Eur J Surg Oncol.

